# Topical Administration of *Gardenia jasminoides* Extract Regulates Th2 Immunity in OVA-Induced Mice

**DOI:** 10.3390/cells12060941

**Published:** 2023-03-20

**Authors:** Sun Haeng Park, Hyun-Kyung Song, Kon-Young Ji, Dong Ho Jung, Seol Jang, Taesoo Kim, Ho Kyoung Kim

**Affiliations:** Herbal Medicine Research Division, Korea Institute of Oriental Medicine (KIOM), Daejeon 305-811, Republic of Korea

**Keywords:** *Gardenia jasminoides* extract, Allergy, Atopic dermatitis, BALB/C mice, cytokines

## Abstract

A key feature of an allergic immune response is a T helper type 2 (Th2)-mediated response with production of allergen-specific IgE antibodies. *Gardenia jasminoides* extract with the crocin removed (GJExCR) has been shown to inhibit IgE-mediated allergic disease. To evaluate the efficacy and mechanism-of-action of this inhibition, GJExCR was used in an ovalbumin (OVA)-induced allergy model in BALB/C mice. Sensitization of BALB/C mice with OVA and aluminum hydroxide was performed on days 1 and 14 by intraperitoneal injection, followed by OVA challenge to the dorsal skin for 2 weeks before removal. Seven days post-challenge, mice were treated with GJExCR topically every day for 11 days. Enzyme-linked immunosorbent assay, flow cytometry analysis, real-time PCR, and western blot were performed to determine IgE and Th2 cytokine levels. Following OVA challenge, Th2 cytokine expression and both total and OVA-specific serum IgE levels increased, of which OVA-specific IgE and Th2 cytokine levels decreased after GJExCR treatment. Flow cytometry analysis revealed that GJExCR treatment decreased CD4+ and CD8+ T cell populations in the spleen and lymph nodes. In addition, treatment with GJExCR downregulated signal transducer and activator of transcription 1 (STAT1) activation and Th2 cytokine levels as compared to control. GJExCR containing geniposide downregulated STAT1 activation in HaCaT cells. These findings demonstrate that GJExCR exerts its anti-allergy effect via inhibition of STAT1 activation, thus regulating the immune response via modulation of Th2 cytokine release and IgE levels. Therefore, we propose GJExCR as a potential treatment for allergic hypersensitivity reactions.

## 1. Introduction

Atopic dermatitis (AD) is a skin allergy mediated by a T helper type 2 cell (Th2)-driven inflammatory response. AD skin lesions are characterized by dermal infiltration of CD4+ T cells and eosinophils along with deposition of eosinophil products and increased skin expression of Th2 cytokines [1]. Specifically, Th2-type cytokines interleukin (IL)-4, IL-5, and IL-13 likely contribute to the pathogenesis of AD [2]. A recent review highlights the functions and characteristics of pathogenic Th2 cells, including pathogenic memory Th2 cell subpopulations during allergic inflammation [3]. Epicutaneous (EC) sensitization leads to a Th2-type immune response, as studies show that ovalbumin (OVA) application to mouse skin results in antigen-specific IgE induction [4]. As a result, OVA is one of the most extensively studied allergens and many studies have reported dose responses, sensitization times, and treatment time points in animal models, including BALB/c mice [5]. Furthermore, EC sensitization induces localized AD in allergen-exposed area in these mice [6].

Th2 cells are crucial for many types of allergic reactions. For example, Th2-related cytokines, IL-4 and IL-10, associate with humoral immunity and anti-inflammatory properties. IL-4-deficient mice clear the helminth, *Nippostrongylus brasiliensis*, more rapidly despite reduced IgE titers [7]. IL-4 is expressed within activated CD4+ T cells in the lymph node, although several studies have shown these cytokine-secreting cells to be a mixed population of Th2 cells [8,9]. CD4+ T cells are also responsible for orchestrating immune responses to pathogens, as well as tolerance to ‘‘self’’ antigens and environmental allergens. To achieve these broad outcomes, activated CD4+ T cells differentiate into T helper cells with specialized functions [10]. Administration of wild-type syngeneic CD4+ T cells, either CD25+ or CD25−, to an Ova-specific T-cell receptor transgenic hemagglutinin-specific B cell receptor knocked-in monoclonal mouse prevents hyper-IgE production. Interestingly, this lack of IgE hyperproduction is not due to initial expansion or contraction responses of the Ova-specific T lymphocytes, but rather from inhibition of their development into IL-4 secreting Th2 lymphocytes [11]. The regulation of mitogen-activated protein kinase (MAPK) and signal transducer and activator of transcription 1 (STAT1) activation may be important in the Th2 related chemokine synthesis, it lead to the migration of Th2 cells into the AD skin lesion [12,13].

*Gardenia jasminoides* extract (GJE), with the crocin carotenoid removed, (GJExCR) has been used in traditional oriental medicine for the treatment of jaundice, fever, hypertension, and skin ulcers [12,14]. Several herbal extracts are reported to have anti-allergic and anti-inflammatory activity by inhibiting degranulation of mast cells by regulating β-Hexosaminidase activity [15,16]. GJExCR shows β-Hexosaminidase activity and possible anti-inflammatory action in mast cells GJE contains geniposide, which inhibits airway inflammation and hyperresponsiveness in mice through p38 MAPK activation [17]. Our previous work has shown that although GJExCR treatment inhibits AD through increasing skin barrier function in *Dermatophagoides farinae* extract (DfE)-induced NC/Nga mice [18], one component of GJE, crocine, has no effect on AD [18,19]. Here, we further investigated the effectiveness of GJE after removal of the crocin and the mechanism of action for GJExCR protection in regulation of CD4+ and CD8+ cells, and the Th2 related cytokine response during the development of an allergic response in OVA-induced mice.

## 2. Materials and Methods

### 2.1. Animals

BALB/c male mice were purchased from Central Lab Animal Inc. (Seoul, Korea) and housed in environmentally controlled, including temperature and light, pathogen-free conditions throughout the experiments. Mice were fed a standard laboratory sterilization diet (Purina 38057, Cargill Agri Purina Inc., Sungnam, Korea), given tap water *ad libitum*, and provided corncob natural bedding material (BioCOB 10384197, Bio System Corporation Pte Ltd., Peninsula Plaza, Singapore). Animals were adapted to this environment for 7 days prior to experiments. All procedures conformed to regulatory standards and were approved by the Institutional Animal Care and Use Committee at Korea Institute of Oriental Medicine (No. 19-015).

### 2.2. OVA-Induced AD Model

Sensitization of BALB/c mice was performed on days 1 and 14 by intraperitoneal injection containing OVA (20 µg) and aluminum hydroxide (2.25 mg). During days 1 to 14, OVA (100 µg in 100 µL of normal saline) or placebo (100 µL of normal saline) was placed on a 1 × 1 cm patch of sterile gauze and then secured to the skin with a transparent, bio-occlusive dressing. The patch was left in place for a 1 week period before removal. This experimental protocol has been previously described [6]. On day 7, mice were randomly divided into treatment groups: either 1% or 3% GJExCR (dissolved in a 4:1 acetone:olive oil solution) or positive control (100 μg tacrolimus with 0.1% protopic ointment). Topical drugs were administered on a daily basis for 14 days on the back and ears of mice starting from day 7. The animal samples, comprising serum and tissue, were obtained from NC/Nga mice that were sacrificed on the final day (Day 20) of the experiment and were subsequently cryogenically stored at −80 °C until analysis.

### 2.3. Preparation of GJExCR

*G. jasminoides* was obtained from Mega Herb, Co. (CheonBuk, Korea). *G. jasminoides* dried plants were extracted with 70% ethanol for 120 min followed by sequential filtration through 5 µm and 1 µm filters. The filtrate was incubated with activated carbon. After filtration through a 1 µm filter, the extract was dried under reduced pressure with a yield of 8.6%.

### 2.4. Automated Hematology Analysis

Automated hematology analysis of EDTA-treated whole blood was determined using a HEMAVET 950 automatic analyzer (DrewScientific, Miami Lakes, FL, USA).

### 2.5. Enzyme-Linked Immunosorbent Assay

The levels of IL-4, IL-5, IL-10, IL-13, and IgE were determined by enzyme-linked immunosorbent assay (ELISA) using specific assay kits (R&D Systems Inc., Minneapolis, MN, USA; Abcam, Cambridge, UK; Shibayagi, Gunma, Japan) according to the manufacturer’s protocol. Absorbance was measured using a Thermo MultiSkan GO Microplate Reader (Thermo Fisher Scientific, Waltham, MA, USA). All experiments were performed in triplicate with at least three biological replicates.

### 2.6. Real-Time PCR

Spleen tissue was homogenized using easy-BLUE reagent and RNA was extracted. cDNA was synthesized by equalizing all samples to the same total amount of RNA and adding a synthetic reagent. Real-time PCR primers, PCR Master Mix (Applied Biosystems, Foster City, CA, USA), and mRNA were loaded onto a MicroAmp Fast 96-well reaction plate. Primers and probes for IL-4 (Mm00445259_m1), IL-5 (Mm00439646_m1), IL-10 (Mm01288386_m1), IL-13 (Mm00434204_m1), and tumor necrosis factor alpha (TNF-α) (Mm004443258_m1) from the TaqMan Gene expression Assay Kit (Applied Biosystems) were quantified using Quantstudio 6 Flex (Applied Biosystems). Cytokine gene expression was normalized to GAPDH (Mm99999915_g1).

### 2.7. Th2 Cell Differentiation

CD4+ naïve T cells were isolated from BALB/c mice using a Naïve CD4+ T cell Isolation Kit (Miltenyi Biotec, Auburn, CA, USA) according to the manufacturer’s protocol. The isolated CD4+ naïve T cells were cultured for 6 days to differentiate into Th2 cells using the CellXVivo Mouse Th2 Cell Differentiation Kit (R&D Systems Inc.) and treated with GJExCR during the experimental periods. After differentiation, the cells were used to analyze the population and activation of Th2 cells.

### 2.8. Flow Cytometry Analysis

Immune cells were isolated from the spleen and lymph node of OVA-induced AD mice. The cells were stained with anti-CD4-FITC and anti-CD8-APC antibodies (BD Biosciences, San Jose, CA, USA). The differentiated Th2 cells were stained with anti-CD3-FITC, anti-CD4-BB700, and anti-GATA3-BV421 (BD Biosciences). After staining, the populations of CD4+ T cells, CD8+ T cells, and Th2 cells were analyzed using a LSRFortessa™ X-20 flow cytometry (BD Biosciences) and FlowJo v. 10 software (FlowJo, Ashland, OR, USA).

### 2.9. Cytokine Secretion Analysis

The differentiated Th2 cells were plated in 96-well plates at density of 1 × 106 cells/mL and treated with GJExCR and Cell Activation Cocktail (R&D systems Inc.; Cat. No. #5476) for 24 h. After incubation, the culture medium of Th2 cells was harvested and cytokine secretion was analyzed using a LEGENDplex™ Mouse Th Cytokine Panel (12-plex) assay kit (BioLegend, San Diego, CA, USA) and LSRFortessa X-20 flow cytometry (BD Biosciences).

### 2.10. Cell Culture

The spontaneously immortalized human keratinocyte cell line (HaCaT) was cultured in Dulbecco’s Modified Eagle’s Medium (DMEM, Gibco BRL, Gaitherburg, MD, USA) with 10% fetal bovine serum, 100 U/mL penicillin, and 100 μg/mL streptomycin at 37 °C in a humidified, 5% CO_2_ atmosphere.

### 2.11. Western Blot Analysis

HaCaT cells were pretreated with GJExCR or geniposide or crocin for 1 h and incubated with or without TNF-α/interferon gamma (IFN-γ) (each 10 ng/mL) for each time. Whole cell lysates were extracted using cold radioimmunoprecipitation assay buffer with protease and phosphatase inhibitors (Biosesang, Seongnam, Korea), respectively. The protein content of the cells was quantified using a BCA kit. Equal amounts of the extracted protein (20 µg) were separated using 4–15% Mini-PROTEAN^®^ TGX™ Precast Protein Gels (Bio-Rad, Hercules, CA, USA) via electrophoresis and then transferred on to Fluoro Trans^®^ polyvinylidene fluoride membrane (Pall Corporation, Dreieich, Germany). The membranes were blocked with 5% skim milk (Sigma, St. Louis, MO, USA) or 3% bovine serum albumin (MP Biomedicals, Irvine, CA, USA) for 2 h, and the primary antibodies (Cell Signaling, Technology Beverly, MA, USA) were incubated with the membrane in blocking solution at 4 °C overnight, followed by incubation with secondary antibodies (Santa Cruz Biotechnology, Texas, USA) at 4 °C for 1 h. ChemiDoc Imaging System (Bio-Rad, Hercules, CA, USA) was used to detect protein expression.

### 2.12. High-Performance Liquid Chromatography Analysis

The high-performance liquid chromatography (HPLC) analysis was performed with a Waters e2695 liquid chromatography system (Waters Co., Milford, MA, USA), equipped with a Waters 2998 photodiode array detector. The Phenomenex Luna C18 column (250 mm × 4.6 mm; particle size 5 μm; Phenomenex, Torrance, CA, USA) was used for chromatographic separation and detected at 260 nm. The mobile phase consisted of 0.1% aqueous acetic acid (A) and 0.1% acetic acid in acetonitrile (B). The elution conditions involved holding the starting mobile phase at 90% A and 10% B and applying a gradient of 50% A and 50% B for 30 min. A wash with acetonitrile was applied for 10 min, followed by equilibration at 90% A and 10% B for 10 min. The flow rate was 1.0 mL/min and the injection volume for all the samples was 20 µL.

### 2.13. Cell Viability

Cells were seeded in 96-well, flat-bottom plates (5 × 10^4^ cells/well) and incubated in the presence of GJExCR or geniposide, crocin for 24 h. MTT solution (0.5 mg/mL) was added to each well for 2 h (5% CO_2_ at 37 °C). Dimethyl sulfoxide (DMSO, 100 μL) was added to solubilize purple formazan crystals. Absorbance at 540 nm was measured by microplate reader (Thermo Fisher Scientific).

### 2.14. Data Analysis

Values are expressed as mean ± standard error of the mean (SEM). Statistical comparisons were performed using unpaired student’s *t*-test and analysis of variance (ANOVA) for repeated measures by Graphpad Prism software version 8.4.3, 2020 (GraphPad Software, LLC. San Diego, CA, USA). Differences were considered significant at *p* < 0.05.

## 3. Results

### 3.1. Phytochemical Components of GJExCR

**Figure 1:**
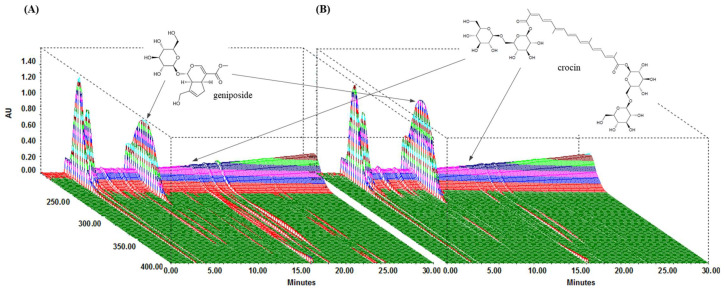
Analysis of GJExCR chemical constituents by HPLC. (**A**) GJE and (**B**) GJExCR.

We performed HPLC analysis of the chemical content of the two compounds, geniposide and crocin, contained within GJE and GJExCR. These compounds were quantified at UV wavelengths of 260 nm, and the three-dimensional HPLC chromatograms for the two samples are shown in Figure 1. Geniposide and crocin were detected in GJE at 9.85 min and 16.15 min, respectively, and in GJE-C at 9.80 min and 16.07 min, respectively. The results for calibration curves are in Table 1, and two compounds showed good linearity (*r*^2^ ≥ 0.9996). In GJE and GJExCR, the geniposide content was detected as 116.41 mg/g and 157.91 mg/g, respectively, whereas the content of crocin was detected as 5.10 mg/g in GJE and 3.52 mg/g in GJE-CR (Table 1 and Figure 1A,B).

### 3.2. GJExCR Regulates Immune Cells in OVA-Induced Mice

Hematological analysis of the mice with OVA-induced allergy showed increased numbers of lymphocytes (LY), basophils (BA), monocytes (MO), esoinophils (EO), and neutrophils (NO) as compared to wildtype (WT). However, mice treated with GJExCR at a concentration of 1 or 3% showed recovery of 25, 60, 76, 54 and 27% LY, BA, MO, EO, NE, respectively (Table 2).

### 3.3. GJExCR Suppresses the Th2 Cytokine Response in OVA-Induced Mice

**Figure 2:**
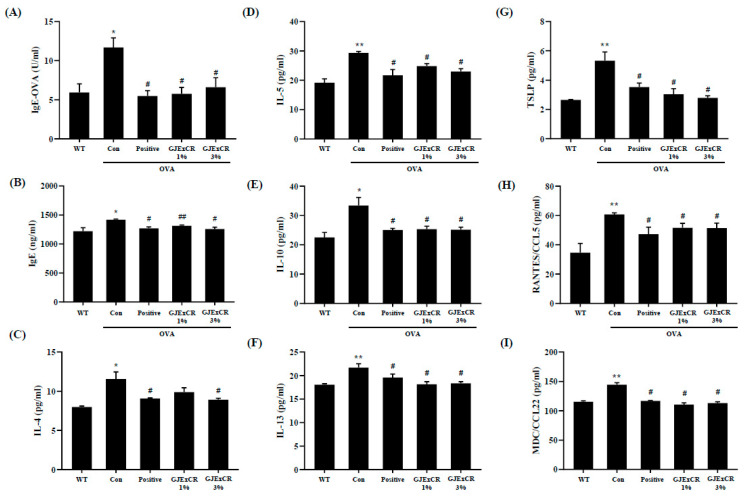
GJExCR treatment significantly attenuates IgE and cytokine levels in the serum of allergic mice. (**A**) Both serum OVA-IgE and (**B**) total serum IgE were evaluated by ELISA. (**C**–**I**) IL-4, IL-5, IL-10, IL-13, TSLP, CCL5, and CCL22 levels in the serum of allergic mice were assessed by ELISA. Data are mean ± SEM. * *p* < 0.05, ** *p* < 0.01 vs. Normal; ^#^ *p* < 0.05, ^##^ *p* < 0.01vs. Control.

GJExCR treatment has been shown to decrease Th2-related cytokine expression as well as IgE levels. To determine if GJExCR treatment regulates the Th2 response during allergy, we assayed serum Th2-related cytokine and chemokine levels in OVA-induced AD mice. In our allergy model without treatment, both IgE and OVA-specific IgE (OVA-IgE) levels, as well as Th2 related cytokine and chemokine levels were increased. However, this increase in OVA-IgE and IgE was attenuated after topical GJExCR treatment (Figure 2A,B). Strikingly, Th2-related cytokines, including IL-4, IL-5, IL-10, IL-13, and chemokines CC chemokine ligand 5 (CCL5), CCL22, and thymic stromal lymphopoietin (TSLP) were significantly suppressed by GJExCR (IgE, *F*(4,14) = 5.56, *p* < 0.001; OVA-IgE, *F*(4,14) = 6.44, *p* < 0.001; IL-4, *F*(4,14) = 7.70, *p* < 0.001; IL-5, *F*(4,14) = 9.54, *p* < 0.001; IL-10, *F*(4,14) = 6.76, *p* < 0.001; IL-13, *F*(4,14) = 6.79, *p* < 0.001; CCL5, *F*(4,14) = 5.28, *p* < 0.001; CCL22, *F*(4,14) = 5.98; TSLP, *F*(4,14) = 10.37, *p* < 0.001, Figure 2C–I).

**Figure 3:**
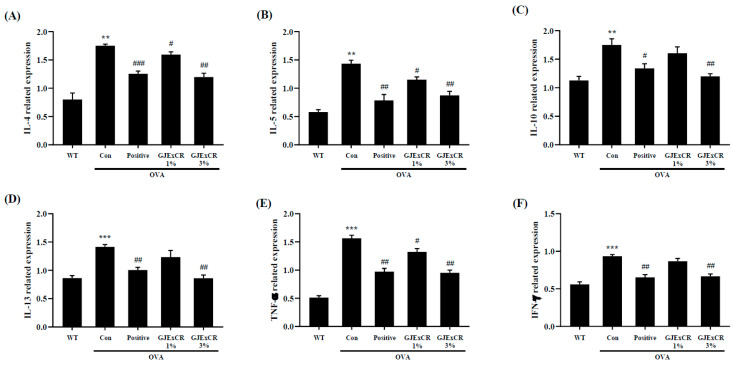
GJExCR treatment dampens Th2-related cytokines and inflammatory factors in the spleen of allergic mice. mRNA expression of (**A**) IL-4, (**B**) IL-5, (**C**) IL-10, (**D**) IL-13, (**E**) TNF-α, and (**F**) IFN-γ in spleen tissue were detected by RT-PCR. Data are mean ± SEM. ** *p* < 0.01, *** *p* < 0.001 vs. Normal; ^#^ *p* < 0.05, ^##^ *p* < 0.01, ^###^ *p* < 0.001 vs. Control.

IL-10, IL-13, and TNF-α expression were found to be higher in OVA-induced mice. Similar to the serum results, GJExCR topical treatment significantly attenuated the increased cytokine expression at the mRNA level after OVA challenge (IL-4, *F*(4,14) = 23.29, *p* < 0.001; IL-5, *F*(4,14) = 23.41, *p* < 0.001; IL-10, *F*(4,14) = 9.18, *p* < 0.001; IL-13, *F*(4,14) = 12.36, *p* < 0.001; TNF-α, *F*(4,29) = 63.57 *p* < 0.001; IFN-γ, *F*(4,29) = 23.65, *p* < 0.001, Figure 3A–F). Collectively, these data indicate that the Th2 response in response to OVA-induced AD can be regulated by GJExCR treatment.

### 3.4. GJExCR Treatment Restores CD4+ and CD8+ T Cell Populations in OVA-Induced AD Mice

**Figure 4:**
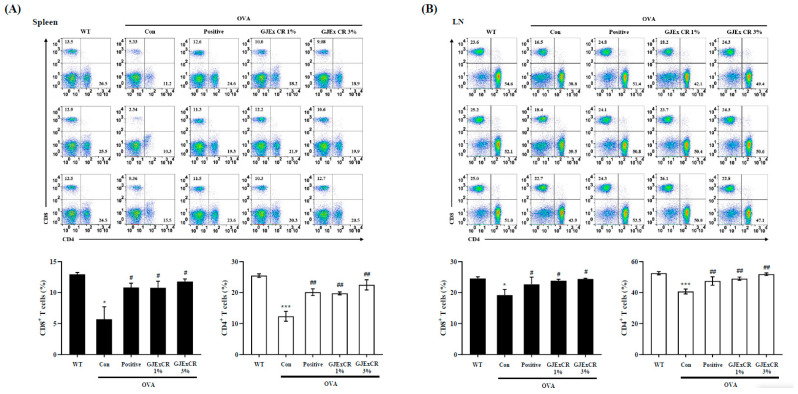
GJExCR treatment restores CD4+ and CD8+ T cell populations in the spleen and lymph node of OVA-induced mice. Isolated cells were analyzed by flow cytometry. Dot plots show the population of CD4+ and CD8+ T cells in (**A**) spleen and (**B**) lymph node. Data are mean ± SEM. * *p* < 0.05, *** *p* < 0.001 vs. WT; ^##^ *p* < 0.01; ^#^ *p* < 0.05 vs. AD.

To determine the beneficial effects of GJExCR on the OVA-induced AD mice, we investigated CD4+ and CD8+ T cell populations within the spleen and lymph node. The percentages of both CD4+ and CD8+ T cells were significantly decreased in the spleen and lymph node of OVA-induced AD mice compared to WT. Interestingly, this observed decrease was not restored by administration of the GJExCR (Spleen CD4+ T Cells, *F*(4,14) = 17.23, *p* < 0.001; Spleen CD8+ T Cells, *F*(4,14) = 5.41, *p* < 0.001; Lymph node CD4+ T Cells, *F*(4,14) = 8.81, *p* < 0.001; Lymph node CD8+ T Cells, *F*(4,14) = 2.37, *p* < 0.001, Figure 4A,B).

### 3.5. GJExCR Suppresses the Th2 Cytokine Response in OVA-Induced Mice

**Figure 5:**
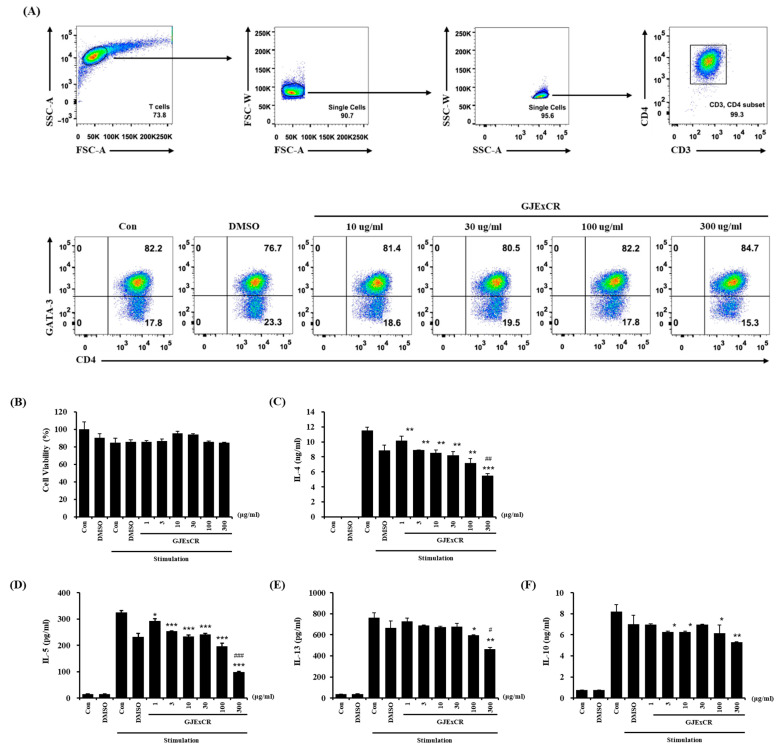
GJExCR treatment inhibits the activation of Th2 cells. (**A**) Flow cytometry analysis of the CD3+CD4+GATA3+ Th2 cell population. (**B**) Measurement of cell viability of differentiated Th2 cells after treatment with GJExCR and cell activation cocktail for 24 h. Analysis of the secretion of Th2 cytokines including (**C**) IL-4, (**D**) IL-5, (**E**) IL-13, and (**F**) IL-10 was performed on the culture medium of harvested Th2 cells stimulated by the GJExCR and activation cocktail. Data are presented as the mean ± SD. * *p* < 0.05, ** *p* < 0.01, *** *p* < 0.001 vs. stimulation control; ^#^ *p* < 0.05, ^##^ *p* < 0.01; ^###^ *p* < 0.001 vs. DMSO stimulation control.

To investigate the mechanism of the restoring effects of GJExCR on T cell populations, we analyzed the changes in differentiation in Th2 cells with or without GJExCR treatment. The population of CD3+CD4+GATA3+ Th2 cells, observed at 82.2% after differentiating Th2 cells from naïve CD4+ T cells, was unchanged with treatment of GJExCR during differentiation (Figure 5A). Next, we evaluated whether the treatment of GJExCR affected the activation of Th2 cells after differentiation. Importantly, no cytotoxicity of GJExCR was observed with increasing concentrations (Figure 5B). Interestingly, the secretion of Th2 cytokines including IL-4, IL-5, IL-13, and IL-10 was significantly suppressed by the treatment of GJExCR in a dose-dependent manner compared to stimulation controls (Figure 5C–F). These results suggest that the treatment of GJExCR ameliorates OVA-induced AD in mice through the inhibition of the activation of Th2 cells, rather than their differentiation.

### 3.6. GJExCR Suppresses p38 MAPK/STA1 Activation in HaCaT Cells

**Figure 6:**
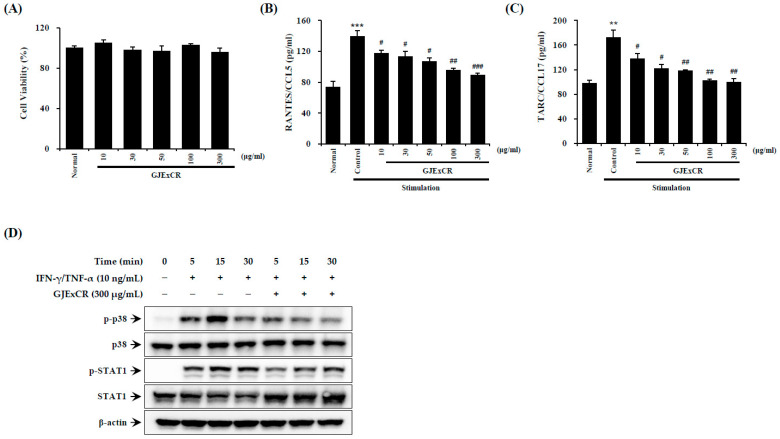
GJExCR treatment reduces p38/STAT1 activation in HaCaT cells. The (**A**) cell viability, the levels of (**B**) CCL5 and (**C**) CCL17 chemokines were measured by ELISA. (**D**) The protein expression of p-p38, p38, p-STAT1, STAT1 in HaCaT cells was detected by western blot. Data are mean ± SEM. ** *p* < 0.01, *** *p* < 0.001 vs. stimulation control; ^#^ *p* < 0.05, ^##^ *p* < 0.01, ^###^ *p* < 0.001 vs. stimulation control.

Inflammatory reactions aggravate symptoms such as skin inflammation in AD. Therefore, we sought to investigate the effect of GJExCR treatment on inflammatory activation in HaCaT cells. As shown by ELISA on TNF-α- and IFN-γ-treated HaCaT cells, GJExCR treatment suppressed p38 MAPK and STAT1 activation, as measured by p38 and STAT1 phosphorylation. Additionally, GJExCR significantly suppressed levels of chemotaxis factors, CCL5 and CCL17, in these HaCaT cells (CCL5 *F*(6,20) = 22.67, *p* < 0.00; CCL17 *F*(6,20) = 21.97, *p* < 0.0011, Figure 6A–D).

### 3.7. GJExCR Contains a Compound That Suppresses the STAT1 Activation in HaCaT Cells

**Figure 7:**
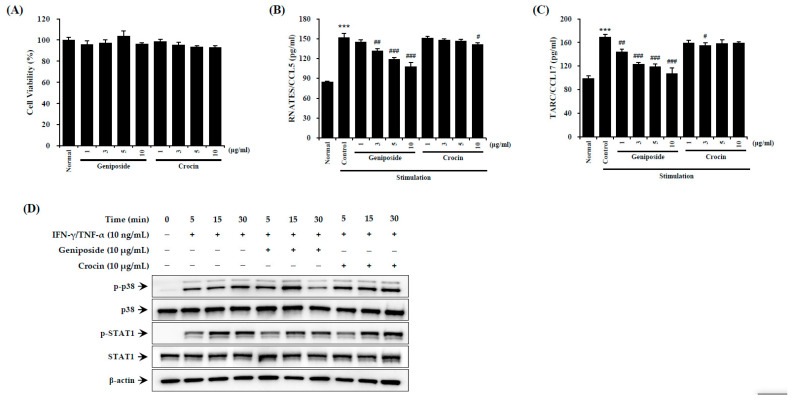
Geniposide reduces p38/STAT1 activation in HaCaT cells. (**A**) Cell viability and the levels of (**B**) CCL5 and (**C**) CCL17 chemokines were measured by ELISA. (**D**) The protein expression in HaCaT cells was detected by western blot. Data are mean ± SEM. *** *p* < 0.001 vs. stimulation control; ^#^ *p* < 0.05, ^##^ *p* < 0.01, ^###^ *p* < 0.001 vs. stimulation control.

To investigate the effects of the compound geniposide, found within GJExCR, on AD-like inflammation, we measured inflammatory factors using ELISA and western blotting in TNF-α- and IFN-γ-treated HaCaT cells. Activation of STAT1 was significantly reduced by geniposide in HaCaT cells. CCL5 and CCL17 were also suppressed by geniposide, while crocin had no effect on either CCL5 or CCL17 expression (CCL5 *F*(9,29) = 102.19, *p* < 0.001; CCL17 *F*(9,29) = 34.03, *p* < 0.001, Figure 7A–D).

## 4. Discussion

Although AD pathogenesis has specific immune and inflammatory mechanisms, the general characteristics include excessive infiltration of inflammatory cells, such as lymphocytes, macrophages, and granulated mast cells into skin lesions, eosinophilia in peripheral blood, and high levels of serum IgE [20]. Importantly, the central pathophysiology in AD is the generation of allergen-specific IgE, most of which is Th2-mediated. We previously reported that GJExCR treatment improved AD skin symptoms by enhancing skin barrier factors in DfE-induced NC/Nga mice [18]. In the present study, we demonstrated the effect of GJExCR treatment on an OVA-induced allergic reaction.

Release of proinflammatory IgE from plasma cells, mast cells, and eosinophils is important in OVA-induced allergy, as levels of serum IgE increase after antigen-specific IgE stimulation via intranasal challenge with OVA [21]. Here, we show that GJExCR treatment reduced the Th2-mediated immune response in OVA-induced allergy, including dampening of IL-4, IL-5, IL-10, IL-13, TNF-α, INF-γ, chemokine, and IgE expression, in both serum and spleens. GJExCR treatment dampened the allergy response via suppression of proinflammatory factor expression, which ultimately inhibited the Th2 response. Consistent with this finding, a recent study attenuated an allergic response by modulation of Th1/Th2 cytokine and IgE levels, which inhibited the proinflammatory response [22]. Interestingly, Th2-dominant immune response results from an imbalance between Th2 and Th1 actions, which plays a crucial role in the development of AD [23].

We also investigated CD4+ T helper cells in OVA-induced allergy response. CD4+ T cells play a pivotal role in infection, inflammation, and autoimmunity through cytokine release, including IL-4, IL-13, and IL-17, which induces a cascade of reactions that act against allergens [24,25]. GJExCR treatment reduced CD4+ and CD8+ T cell populations in lymph nodes and spleen. OVA-specific CD4+ and CD8+ T cell proliferation appeared first in lymph nodes and later in the spleen, suggesting a possible migration of activated cells from the site of induction into systemic circulation. CD4+ T cells support the generation of CD8+ T cells in this system. Interestingly, we observed a significant correlation between the percentages of proliferating CD4+ and CD8+ T cells for each animal [26]. Previous studies found that vaccines able to activate both CD4+ and CD8+ T cell responses result in better immunity in patients [27]. Naïve CD4+ T cells differentiate into a number of specialized Th cell subsets. In addition, repression of STAT1 decreased the effects of downstream STAT1-dependent inflammatory mediators, including secondary effects on inflammatory cells, including Th cell cytokine and chemokine production, Ag-specific Ig levels, and mucous cell metaplasia in OVA induced allergy model [28].

Reports show that IL-4 induces the differentiation of naïve CD4+ T cells to Th2 cells via binding to the IL-4 receptor. CD4+ T cells can be subdivided into Th1, Th2, Th17, and Treg subsets on the basis of their pattern of cytokine production [29]. In our study, STAT1 increased within HaCaT cells after TNF-α/IFN-γ stimulation. However, this STAT1 increase was suppressed after GJExCR treatment, as was expression of other Th2 type chemokines and cytokines. IL-4 and IL-13, Th2 cytokines, decrease the expression of epidermal differentiation complex genes filaggrin, loricrin, and involucrin in differentiated primary human keratinocytes [30]. Interestingly, we previously reported that GJExCR treatment strengthened skin barriers in DfE induced NC/Nga mice [18]. Furthermore, the GJExCR compound geniposide had the same effect as GJExCR treatment, but crocin had no effect on the immune response.

Long-term use of topical corticosteroids, particularly those of high potency, cause common side effects. Although many studies have examined the use of herbal medicines for the treatment of individual or multiple health conditions [31], insufficient evidence currently exist to support the use of herbs for the treatment of AD [32]. Our results support GJExCR as a potential anti-allergic agent for the clinical management of AD.

## 5. Conclusions

This investigation demonstrated the anti-allergic potential of GJExCR against OVA-induced AD. GJExCR exerted its effect by inhibition of allergen-induced hypersensitivity via inhibition of p38 MAPK/STAT1 activation, resulting in the regulation of Th2 response and decreased release of CD4+ T cells, CD8+ T cells, and IgE.

## Figures and Tables

**Table 1:** Calibration curves and contents of the two compounds in GJE and GJE-C.

Compound	Regression Equation				Content (mg/g)	
	Linear Range (µg/mL)	Slope	Intercept	*r* ^2^	GJE (Mean ± SD)	GJE-C (Mean ± SD)
Geniposide	3.13–200.00	10868	16900	0.9996	116.41 ± 0.21	157.91 ± 0.52
Crocin	3.13–200.00	11893	−3407.8	0.9999	5.10 ± 0.05	3.52 ± 0.02

**Table 2:** Hematological analysis.

Parameters	Group				
	WT	Control	Positive	GJExCR 1%	GJExCR 3%
LY (%)	34.6 ± 2.37	84.8 ± 1.59 ***	52.5 ± 0.39 ^###^	76.6 ± 0.85 ^##^	63.4 ± 0.57 ^###^
BA (%)	0.3 ± 0.11	3.7 ± 0.12 ***	0.8 ± 0.11 ^###^	1.7 ± 0.08 ^###^	1.3 ± 0.11 ^###^
MO (%)	2.0 ± 0.29	6.1 ± 0.76 **	2.2 ± 0.08 ^##^	4.0 ± 0.49 ^#^	2.5 ± 0.29 ^##^
EO (%)	0.2 ± 0.16	3.5 ± 0.32 ***	0.7 ± 0.15 ^###^	2.4 ± 0.43	1.3 ± 0.10 ^###^
NE (%)	40.9 ± 0.84	69.4 ± 0.78 ***	46.7 ± 2.70 ^###^	59.4 ± 0.70 ^###^	50.5 ± 2.02 ^###^

Values represent mean ± SEM. ** *p* < 0.05, *** *p* < 0.001 vs. WT; ^#^ *p* < 0.05, ^##^ *p* < 0.01, ^###^ *p* < 0.001 vs. Control. LY (Lymphocyte); BA (Basophil); MO (Monocytes); EO (Eosinophils); NE (Neutrophils).

## Data Availability

The data presented in this study are available on request from the corresponding author.
